# How mathematics homework types relate to mathematics academic performance via interest? A chain mediation analysis in the Chinese context

**DOI:** 10.3389/fpsyg.2026.1815534

**Published:** 2026-08-14

**Authors:** Mucheng Zhang, Yueyang Shao, Jian Liu

**Affiliations:** 1 Collaborative Innovation Center of Assessment Toward Basic Education Quality, Beijing Normal University, Beijing, China; 2 China Education Innovation Institute, Beijing Normal University, Zhuhai, China; 3 Joint Education Institute of Zhejiang Normal University and University of Kansas, Zhejiang Normal University, Jinhua, China

**Keywords:** homework interest, homework types, large scale test, learning interest, mathematics academic performance

## Abstract

The aim of this study is to investigate the relationship between eighth-grade students’ mathematics homework types and their combinations and their Mathematics Academic Performance (MAP) in the context of China’s “Double Reduction” policy, emphasizing the chain mediating role of mathematics homework interest and learning interest. A total of 18,202 eighth-grade students completed a cross-sectional survey administered in a mixed online–offline mode. We adopted a mediation analysis method with multicategorical independent variables, using Practice Homework (PH) as the reference category. The results showed that homework combinations containing Test-Preparation Homework (TPH) tend to improve MAP through direct effects, while homework combinations including Applied/Project-Based Homework (APH)—in particular PH + APH—are more likely to enhance students’ interest and improve MAP indirectly. In addition, homework interest and learning interest mediated the link between specific homework types (and their combinations) and MAP, primarily through a sequential chain mediation pathway in which homework interest predicted learning interest, which in turn predicted MAP. These findings clarify the mechanisms through which homework design—considering both types and combinations—relates to students’ MAP in a “Double Reduction” policy context, offering practical guidance for educators on assigning homework that enhances learning motivation and academic performance.

## Introduction

Influenced by the Confucian heritage culture, students in China and other East Asian systems have long achieved outstanding results in mathematics and science, often accompanied by excessive academic pressure (Ho and Hau, 2010). Within Confucian educational traditions, academic achievement is widely regarded as a primary pathway to individual success and social mobility. Consequently, sustained effort and high academic expectations are strongly emphasized by families and schools, often resulting in substantial homework demands and extended study time (Li, 2002). Evidence links such pressure to heavy homework loads, which elevate cognitive load and fatigue and, in turn, dampen students’ interest (Guo et al., 2024). To alleviate students’ burdens, China introduced the “Double Reduction” policy in 2021. The reform sought to reduce homework pressure and strictly curtail private academic tutoring for Grades 1–9 (Qian et al., 2023). Widely regarded as the most extensive and influential workload-reduction initiative in the country’s history (Xue and Li, 2023), the policy explicitly emphasized high-quality homework and called for the elimination of repetitive, mechanical, and punitive homework. This signaled a shift in policy attention from the quantity of homework to its quality and types—that is, what kinds of tasks best support learning. Because homework quality is difficult to define or measure in the abstract (Xu et al., 2021), researchers have increasingly examined observable characteristics of homework design, such as homework purpose, task characteristics and homework type, to understand variation in homework quality and its influence on student learning (Cooper, 1989; Rosário et al., 2019).

While prior studies generally report a positive association between homework and academic performance (Chin et al., 2022; Maltese et al., 2012), far less is known about the mechanisms through which homework types shape learning outcomes. In particular, it remains unclear whether the association operates via a sequential motivational route—from homework interest to broader learning interest and, in turn, to academic performance.

We focus on mathematics for both substantive and practical reasons. Mathematics occupies a foundational position in compulsory education and typically accounts for a large share of students’ homework; consequently, the most visible homework adjustments under the “Double Reduction” reform have occurred in this subject. Against this backdrop, it is essential to identify the factors that predict mathematics academic performance under the reform and to clarify the underlying processes. Among potential predictors, homework is the most directly targeted by the policy. Interest develops along a continuum from situational (task-triggered) to stable individual interest in a broader domain (Hidi and Renninger, 2006). Homework interest, as a response to a recurring task, is situational, whereas learning interest is a general mathematical orientation. Thus, homework interest likely precedes learning interest, supporting their testing as sequential mediators. Accordingly, we examine whether, within the “Double Reduction” context, homework types are indirectly associated with mathematics academic performance through a sequential route of homework interest and learning interest.

## Literature review

### Mathematics homework types and mathematics academic performance

According to functional theories of homework (Epstein and Van Voorhis, 2001; Maharaj-Sharma and Sharma, 2016), homework typically serves three purposes: consolidation and preparation, examination practice, and application and engagement. This tri-partite distinction has been validated across educational contexts (Rosário et al., 2015; Trautwein, 2007). In the Chinese context specifically, evidence indicates that written mathematics homework remains dominated by problem-sets (Wang et al., 2016), while non-written assignments increasingly incorporate practical problem-solving (Ministry of Education of People’s Republic of China, 2022). Building on distinctions and consistent with prior classifications of homework purpose in mathematics education (e.g., Rosário et al., 2015), we classify mathematics homework into three categories: Practice Homework (PH), Test-Preparation Homework (TPH), and Applied/Project-Based Homework (APH) (Table 1). These categories provide a theoretically grounded framework for examining how different homework designs are associated with students’ academic outcomes. While the relationship between time spent on homework and academic performance has received extensive attention (Chin et al., 2022; Maltese et al., 2012), Trautwein (2007) highlighted that the type of homework also matters for academic performance. This effect need not operate solely through cognitive channels; homework types that differ in format and purpose may also elicit different levels of student interest and engagement, which can further shape achievement. For example, some evidence suggests that APH was more related to mathematics achievement than PH and TPH (Rosário et al., 2015). Consequently, this study posits the first hypothesis:

**Table 1:** Homework types in China’s mathematics education.

Homework Type	Format	Purpose	Example
Practice Homework (PH)	Written	Consolidation and preparation	Textbook problem sets/review exercises
Test-Preparation Homework (TPH)	Written	Examination practice	Simulated/previous tests
Applied/Project-Based Homework (APH)	Non-written	Application and engagement	Designing a parking lot/investigating local air pollution

Hypothesis 1. Different mathematics homework types and their combinations predict mathematics academic performance of eighth-grade students differently.

### The mediating role of mathematics homework interest

Grounded in intrinsic motivation theory, interest drives spontaneous learning and facilitates competence development (Deci and Ryan, 2013). Prior research clarifies the mechanisms by which individual interest shapes academic development. One account argues that interest promotes behavioral investment—students with stronger interest devote more efforts and time to learning activities, thereby improving academic performance (Lee et al., 2014; Trautwein et al., 2015; Harackiewicz et al., 2016). A second account emphasizes the role of interest in improving the quality of learning, including concentration, persistence, and depth of processing (Li and Yang, 2016; Hidi et al., 2004; Schiefele, 1998; Alexander and Murphy, 1998) To examine how mathematics homework types and their combinations are related to students’ mathematics academic performance, we therefore conceptualize interest as a potential mediator and distinguish between homework interest and learning interest.

Homework interest refers to students’ intrinsic feelings, attitudes, and motivation toward homework—their direct interest in the task itself (Xu, 2008; Xu, 2021). Classic work urged researchers to treat homework interest as distinct from other interests and to study it as an independent construct (Cooper et al., 1998), a point echoed in more recent studies (Suárez et al., 2019; Huang, 2022). Moreover, homework interest tends to be positively associated with academic performance (Cooper et al., 1998; Trautwein, 2007; Xu, 2008; Suárez et al., 2019). For example, when comparing the predictive powers of students’ homework cognitions, attitudes, and motivations for engagement and performance, Suárez et al. (2019) found that intrinsic motivation towards homework was the strongest positive predictor of academic development. Existing research also indicates that homework type is an antecedent of homework interest: different types can directly affect students’ interest—for instance, students often find practical tasks more engaging (Shernoff et al., 2003; Huang, 2022). Building on evidence linking homework types to homework interest, and homework interest to academic performance, this study posited the second hypothesis:

Hypothesis 2. Mathematics homework interest serves as a mediator between mathematics homework types (and their combinations) and mathematics academic performance, and the size of this mediating effect varies across homework types and combinations.

### The mediating role of mathematics learning interest

Beyond homework interest, learning interest is a second mediator linking homework types (and their combinations) with mathematics academic performance. Mathematics learning interest includes both trait-like interest in mathematics—habitual, relatively stable personal interest that develops over time (Frenzel et al., 2010)—and state interest in mathematics, which is situationally triggered at a particular moment (Hidi, 1990; Schiefele, 1991). In this study we focus on the more stable, trait-like interest in mathematics.

There is ample evidence that learning interest is a strong predictor of academic performance (Köller et al., 2001; Hattie, 2009). At the same time, homework—an obligatory activity that consumes students’ out-of-school time—can directly shape students’ learning interest (Zhang, 2019; Waldspurger, 2022). Notably, when homework is predominantly written assignments and drill-oriented, it may depress learning interest (Kaur, 2011). Hence, different homework types are likely to have different effects on students’ learning interest. Considering the link between homework types and learning interest, and between learning interest and academic performance, this study posited the third hypothesis:

Hypothesis 3. Mathematics learning interest serves as a mediator between mathematics homework types (and their combinations) and mathematics academic performance, and the size of this mediating effect varies across homework types and combinations.

### The relationship between mathematics homework interest and mathematics learning interest

When examining how homework types relate to academic performance, it is important to distinguish between homework interest and learning interest. First, they differ in temporal persistence. Homework interest concerns students’ liking for the homework task itself and can be immediate and short-lived (Xu, 2008). By contrast, mathematics learning interest is relatively long-term and stable (Frenzel et al., 2010). For example, Cooper (1989) characterizes learning interest as a “long-term factor” when discussing how homework affects students’ motivation. Second, they differ in directness. In outlining the development of students’ learning interest, Ainley (2006) argues that when learners lack interest in a subject, making the task itself interesting is crucial to first engage them in the learning activity. This implies that homework types may first shape homework interest, which then fosters learning interest—an ordering supported by prior work (Kralovec and Buell, 2001; Trautwein and Koller, 2003). Accordingly, this study proposed the fourth hypothesis:

Hypothesis 4. Mathematics homework types (and their combinations) are indirectly associated with mathematics academic performance through the chain mediation of Mathematics Homework Interest and Mathematics Learning Interest; moreover, the magnitude of this chain mediation varies across homework types and combinations.

**Figure 1:**
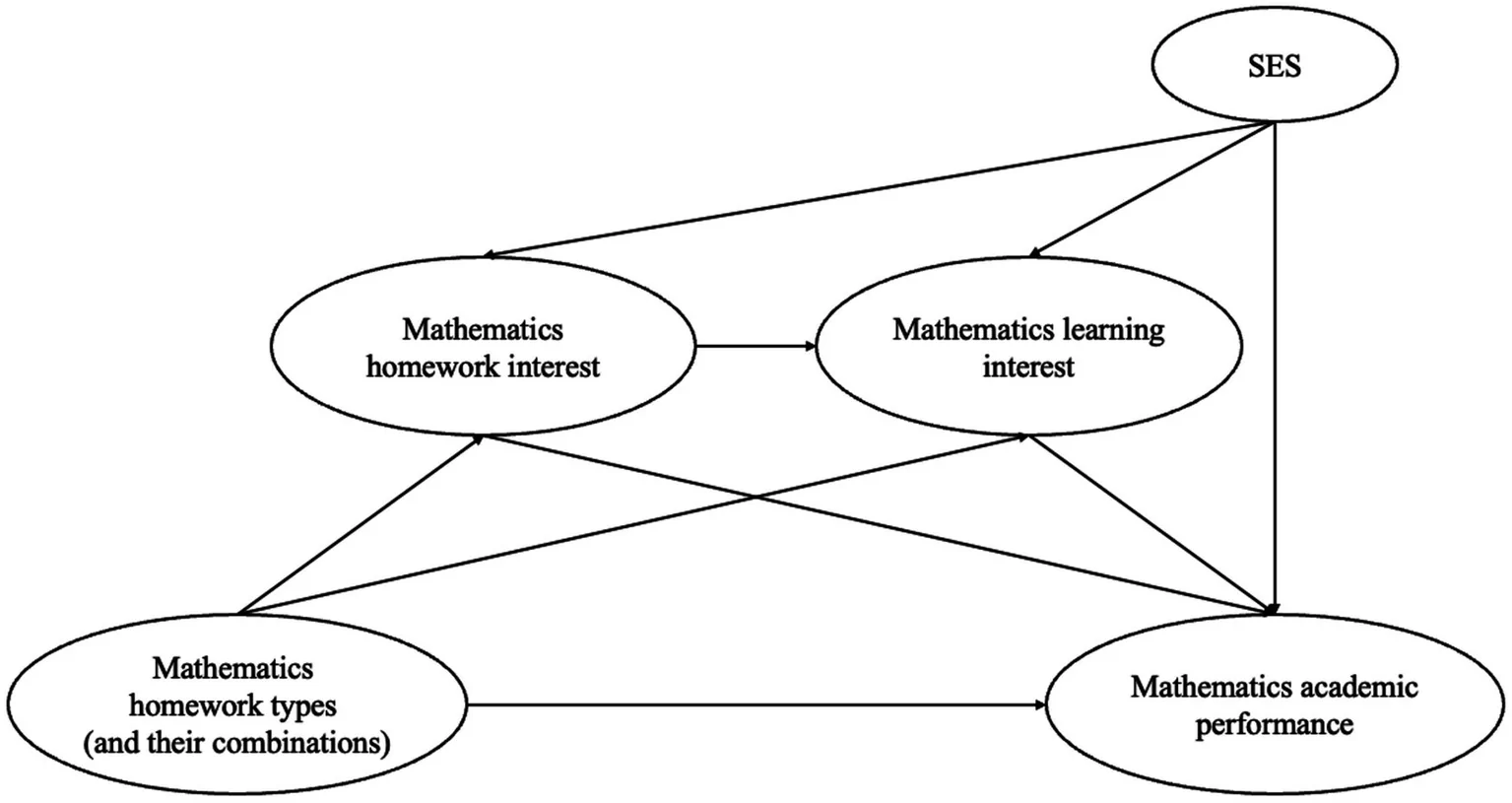
The hypothesised model.

In summary, this study aims to explore the relationship between mathematics homework types (and their combinations) and mathematics academic performance, and examines the mechanisms through which mathematics homework interest and mathematics learning interest operate. Given that students’ socioeconomic status (SES) is commonly treated as a necessary control variable (Agasisti et al., 2018), we include SES as a covariate in the model (see Figure 1). The findings will enrich our understanding of how different homework types (and their combinations) relate to students’ academic performance and will offer practical guidance for designing higher-quality mathematics homework.

## Methods

### Participants and procedure

Data for this cross-sectional study were drawn from the Regional Assessment of Education Quality of the Collaborative Innovation Centre of Assessment towards Basic Education Quality (RAEQ–CICA-BEQ). RAEQ is a division within CICA-BEQ that provides education-quality assessment services for specific regions, cities, and provinces in China. RAEQ-CICA-BEQ adopts probability-proportional-to-size (PPS) sampling design to obtain a representative sample of students from a city in southern China (D city). Firstly, when selecting schools, two variables are mainly considered: the nature of the school (public or private) and the level of development of the school, while also taking into account the size of the school and the administrative region to select school samples proportionally; Ultimately, a total of 188 schools were included. Secondly, within each selected school, the number of students to be tested is determined based on the size of the eighth-grade students in each school. For schools with less than 60 eighth grade students, all eighth-grade students will be included to ensure sample integrity. The students first completed a paper and pencil math academic performance test and then completed an online questionnaire survey organized and implemented by RAEQ. In order to protect the privacy of participating schools and students, the analysis data provided to researchers has been de identified. In total, 18,202 eighth-grade students participated in the assessment (42.5% girls; 57.5% boys).

### Instruments

#### Mathematics homework types (MHT)

RAEQ–CICA-BEQ has developed a multiple-choice question to investigate the types of math assignments students have Firstly, a review was conducted on the classification research of the functions and purposes of mathematical homework, such as the previously mentioned ones consolidation and preparation, examination practice and application and engagement, to build the initial framework. Secondly, we conducted semi-structured interviews with mathematics teachers and students to identify common types and forms of homework in Chinese middle school mathematics teaching and learning. Based on literature and interview results, we have identified three major types of mathematics assignments. Subsequently, we invited mathematics education experts to review the question items to verify the appropriateness and clarity of the description and examples. The stem was: *“Typically, what types of mathematics homework does your teacher assign?”* Response options aligned with our three categories: Practice Homework (PH) (e.g., textbook problem sets, review exercises), Test-Preparation Homework (TPH) (e.g., past tests, simulated tests), and Applied/Project-Based Homework (APH) (e.g., using mathematics to solve real-life problems like designing a parking lot; making handcrafts/models). Because multiple selections were permitted, each option was coded 1 = selected and 0 = not selected, and mutually exclusive combination patterns were derived for analysis (PH only; TPH only; APH only; PH + TPH; PH + APH; TPH + APH; PH + TPH + APH). These indicators and combinations were then used to examine how homework types and their combinations relate to mathematics academic performance, consistent with our theoretical framework.

#### Mathematics homework interest (MHI)

Mathematics homework interest was measured using the homework-interest instrument employed by Xu (2008). The scale comprised three items; for example: *“In your opinion, most of the time, mathematics homework is …”* with response options *“very boring,” “boring,” “neither boring nor interesting,” “interesting,” and “very interesting.”* The scale demonstrated excellent internal consistency (Cronbach’s *α* = 0.93). For analysis, we used the mean score across the three items, with higher values indicating greater homework interest.

#### Mathematics learning interest (MLI)

Mathematics learning interest was assessed with a four-item, five-point Likert scale adapted from the PISA student questionnaire (OECD, 2009). Example items include: *“I enjoy reading materials about mathematics”* and *“I enjoy attending mathematics classes.”* Students indicated their agreement from 1 = strongly disagree to 5 = strongly agree. The scale showed excellent internal consistency (Cronbach’s *α* = 0.95). For analysis, we used the mean score, with higher values reflecting greater learning interest.

#### Mathematics academic performance (MAP)

Mathematics academic performance was assessed with a paper-and-pencil test developed by RAEQ–CICA-BEQ. Aligned with the national curriculum standards for compulsory education (Ministry of Education of the People’s Republic of China, 2011), the test targeted mathematical literacy and provided a comprehensive evaluation of content knowledge and skills in numbers and algebra, geometry and figures, statistics and probability, and integrated/practical applications. The instrument demonstrated strong psychometric properties (Cronbach’s α = 0.892; CFI = 0.942; TLI = 0.937; RMSEA = 0.023; SRMR = 0.034). For analysis, we computed each student’s proportion-correct on the mathematics test and rescaled it to a 0–100 metric (i.e., proportion × 100).

#### Socioeconomic status (SES)

Socioeconomic status (SES) was assessed following the PISA approach (OECD, 2009). Three indicators were used: (a) parents’ occupations, (b) highest parental education, and (c) household possessions, including a private bedroom, a family car, a bathroom equipped with a bathtub or shower, a personal computer or tablet for studying and homework, a quiet study space, and musical instruments (e.g., guitar or piano). The three items formed a composite with acceptable internal consistency (Cronbach’s α = 0.648). For analysis, we computed the mean score across the three indicators, with higher values indicating higher SES.

### Data analysis

The data analysis of this study consists of three steps: (1) data description; (2) correlation analysis; and (3) mediation analysis.

In the first step, the study first cleaned the raw data. Given that the missing rate of each variable was relatively low (< 3%), the listwise deletion method was adopted for data cleaning, and 98% of the cases were retained for subsequent analysis. Secondly, we examined the proportion of students choosing the seven types of mathematics homework and their combinations. Since the proportion of students who chose either “APH only” or “TPH + APH” was less than 1%, resulting in insufficient statistical power and unstable parameter estimates, these two were excluded from subsequent analyses in this study. The final sample size for the actual analysis was 17,664 participants. Specifically, among the retained categories, PH accounted for 31.8%, TPH for 7.9%, PH + TPH for 51.2%, PH + APH for 3.0%, and PH + TPH + APH for 3.2% of the students.

In the second step, SPSS 25.0 was used to conduct descriptive statistics and zero-order correlation analysis.

In the third step, we followed the mediation analysis procedures involving a multicategorical independent variable described by Hayes and Preacher (2014). We treated the multicategorical homework predictor with indicator coding, using Practice Homework (PH) as the reference (coded 0 on all indicators). Four binary indicators captured the remaining patterns:

$D_{1}=$ TPH (1 = TPH only), $D_{2}=$ PH + TPH, $D_{3}=$ PH + APH, $D_{4}=$ PH + TPH + APH.

The serial mediation model specified three regression equations:

$$\begin{array}{cc} \mathrm{MHI} & =i_{1}+\gamma_{1}\;\mathrm{SES}+\alpha_{11}D_{1}+\alpha_{12}D_{2}+\alpha_{13}D_{3}+\alpha_{14}D_{4}+\varepsilon_{1},\\ \mathrm{MLI} & =i_{2}+\gamma_{2}\;\mathrm{SES}+d\;\mathrm{MHI}+\alpha_{21}D_{1}+\alpha_{22}D_{2}+\alpha_{23}D_{3}+\alpha_{24}D_{4}+\varepsilon_{2},\\ \mathrm{MAP} & =i_{3}+\gamma_{3}\;\mathrm{SES}+b_{1}\;\mathrm{MHI}+b_{2}\;\mathrm{MLI}+c_{1}^{\prime }D_{1}+c_{2}^{\prime }D_{2}+c_{3}^{\prime }D_{3}+c_{4}^{\prime }D_{4}+\varepsilon_{3}. \end{array}$$

Relative (PH-referenced) indirect effects were defined as

$$\begin{array}{ccc} \mathrm{via}\;\mathrm{MHI}: & & \alpha_{1k}b_{1},\\ \mathrm{via}\;\mathrm{MLI}: & & \alpha_{2k}b_{2},\\ \text{serial}: & & \alpha_{1k}\;d\;b_{2}, \end{array}k\in \{1,2,3,4\},$$

with total indirect = sum of the three, and total effect $=c_{k}^{\prime }+\text{total indirect}$ .

All coefficients were estimated in Mplus (ML), with bias-corrected bootstrap CIs (1,000 draws). Because the model is saturated (df = 0), global fit indices are uninformative; inference is based on path estimates and bootstrap intervals. In addition, we used MODEL CONSTRAINT to compute pairwise contrasts among D1–D4 for direct effects, each indirect route, the total indirect, and the total effect, with BC-bootstrap Cis.

## Results

### Descriptive statistics and correlations

The means, standard deviations, and zero-order (Pearson) correlations are reported in Table 2. The four continuous variables—MHI, MLI, SES, and MA—were all positively and significantly correlated. The homework–type indicators are binary and showed non-uniform associations with the other variables. For example, PH and PH + TPH were each significantly related to MHI, MLI, and MA, whereas PH + TPH + APH was significantly related to MHI and MLI but not to MA.

**Table 2:** Descriptive statistics and zero-order correlations amongst the main variables.

Variables	1	2	3	4	5	6	7	8	9
1 PH	1								
2 TPH	−0.200^**^	1							
3 PH + TPH	−0.699^**^	−0.299^**^	1						
4 PH + APH	−0.121^**^	−0.052^**^	−0.181^**^	1					
5 PH + TPH + APH	−0.125^**^	−0.053^**^	−0.187^**^	−0.032^**^	1				
6 MHI	0.039^**^	−0.012	−0.105^**^	0.164^**^	0.033^**^	1			
7 MLI	0.054^**^	−0.011	−0.112^**^	0.152^**^	0.038^**^	0.771^**^	1		
8 SES	−0.104^**^	0.042^**^	0.079^**^	−0.001	0.024^**^	0.111^**^	0.113^**^	1	
9 MAP	−0.150^**^	0.010	0.173^**^	−0.014	−0.014	0.266^**^	0.225^**^	0.289^**^	1
M	0.32	0.08	0.51	0.03	0.03	3.43	3.52	6.28	48.82
SD	0.47	0.27	0.50	0.17	0.18	0.96	1.06	1.07	23.23

** represents *p* < 0.01. PH = Practice Homework; TPH = Test-Preparation Homework; PH + TPH = both PH and TPH selected; PH + APH = PH and Applied/Project-Based Homework selected; PH + TPH + APH = PH, TPH, and APH all selected; MHI = Mathematics Homework Interest; MLI = Mathematics Learning Interest; SES = Socioeconomic Status; MAP = Mathematics Academic Performance.

### Mediation analysis

#### Path estimates (indicator coding)

Table 3 reports non-standardized path coefficients (SEs) from the indicator-coded model using PH as the reference category. Consistent with the bivariate patterns, SES positively predicted MHI (*γ*₁ = 0.108, SE = 0.007, *p* < 0.001) and MAP (γ₃ = 0.052, SE = 0.001, p < 0.001). The proximal–to–distal motivational linkage was strong: MHI → MLI was large and positive (d = 0.840, SE = 0.006, *p* < 0.001); both MHI and MLI, in turn, were positive predictors of MAP (b₁ = 0.055, SE = 0.003, p < 0.001; b₂ = 0.011, SE = 0.002, p < 0.001).

**Table 3:** Estimated coefficients using indicator coding (PH as reference).

Outcome	Coefficient (SE)	Coefficient (SE)	Total effect (SE)
	MHI	MAP (with MHI and MLI)	MAP (total)
Constant	$i_{1}=2.825$ (0.042)***	$i_{3}=-0.117$ (0.010)***	—
D1 (TPH)	$\alpha_{11}=-0.135$ (0.027)***	$c_{1}^{\prime }=0.055$ (0.006)***	$c_{1}=0.045$ (0.007)***
D2 (PH + TPH)	$\alpha_{12}=-0.186$ (0.016)***	$c_{2}^{\prime }=0.093$ (0.004)***	$c_{2}=0.081$ (0.004)***
D3 (PH + APH)	$\alpha_{13}=0.814$ (0.037)***	$c_{3}^{\prime }=-0.025$ (0.010)**	$c_{3}=0.029$ (0.011)**
D4 (PH + TPH + APH)	$\alpha_{14}=0.078$ (0.052)	$c_{4}^{\prime }=0.015$ (0.010)	$c_{4}=0.021$ (0.010)*
SES	$\gamma_{1}=0.108$ (0.007)***	$\gamma_{3}=0.052$ (0.001)***	—
MHI → MLI	—	d=0.840 (0.006)***	—
MHI → MAP	—	$b_{1}=0.055$ (0.003)***	—
MLI → MAP	—	$b_{2}=0.011$ (0.002)***	—

* p<.05 , ** p<.01 , *** p<.001 . D1–D4 are indicator-coded contrasts relative to PH.

Relative to PH, homework combinations showed distinct associations with MHI. TPH (D1) and PH + TPH (D2) were associated with lower MHI (*α*₁₁ = −0.135, SE = 0.027, *p* < 0.001; α₁₂ = −0.186, SE = 0.016, p < 0.001), whereas PH + APH (D3) markedly increased MHI (α₁₃ = 0.814, SE = 0.037, p < 0.001). The coefficient for PH + TPH + APH (D4) was small and not significant (α₁₄ = 0.078, SE = 0.052, ns). Relative to PH, the MLI equation showed: D1 and D2 negative ( −0.044 , SE = 0.020, *p* = 0.026; −0.070 , SE = 0.012, *p* < 0.001), D3 positive ( 0.126 , SE = 0.025, *p* < 0.001), and D4 not significant ( 0.040 , SE = 0.035, ns).

**Figure 2:**
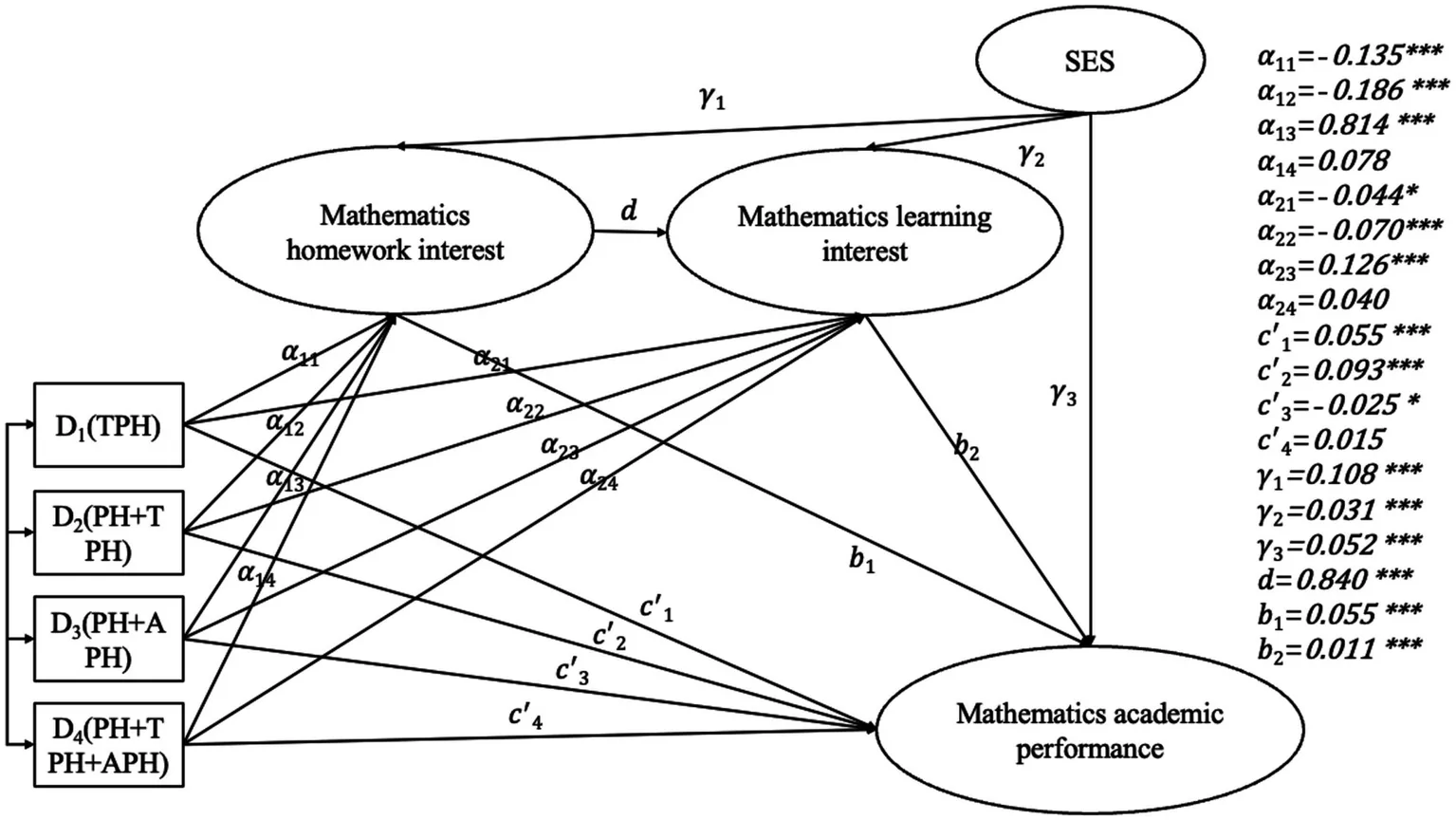
A mediation model in path-diagram form corresponding to a model with a multicategorical independent variable with 5 categories of homework. Note: *p < .05, **p < .01, ***p < .001. D1–D4 are indicator-coded contrasts relative to PH.

In the academic performance equation (controlling SES, MHI, and MLI), TPH and PH + TPH retained positive direct effects on MAP (c′₁ = 0.055, SE = 0.006, *p* < 0.001; c′₂ = 0.093, SE = 0.004, *p* < 0.001), PH + APH showed a small negative direct effect (c′₃ = −0.025, SE = 0.010, *p* < 0.05), and PH + TPH + APH was not significant (c′₄ = 0.015, SE = 0.010, ns). When indirect paths were added (via MHI, via MLI, and serial MHI → MLI), the total effects relative to PH were positive for all four contrasts: TPH c₁ = 0.045 (SE = 0.007, *p* < 0.001), PH + TPH c₂ = 0.081 (SE = 0.004, *p* < 0.001), PH + APH c₃ = 0.029 (SE = 0.011, *p* < 0.01), and PH + TPH + APH c₄ = 0.021 (SE = 0.010, *p* < 0.05) (Figure 2).

#### Relative indirect, total indirect, and total effects

Table 4 summarizes the relative indirect effects of each pattern compared with PH (reference). Two exam-oriented contrasts—TPH and PH + TPH—showed negative total indirect effects (−0.009 and −0.013, both *p* < 0.001). In each case, the decrement was driven primarily by the via-MHI path (−0.007 and −0.010) with an additional negative serial component (−0.001 and −0.002); the via-MLI component was approximately zero for TPH (*p* = 0.050; BC 95% CI includes zero) and small but negative for PH + TPH (−0.001, *p* < 0.001). Despite these suppressive indirect routes, both contrasts retained sizeable positive direct effects (c′ = 0.055 and 0.093), yielding positive total effects relative to PH (0.045 and 0.081).

**Table 4:** Relative indirect and total effects (non-standardized; PH as reference).

Contrast vs. PH	via MHI (***α***_1*k*_*b*_1_)	via MLI (***α***_2*k*_*b*_2_)	Serial (***α***_1*k*_*db*_2_)	Total indirect	Direct $\left(c_{k}^{'}\right)$	Total effect
TPH (D1)	−0.007 (0.002)***	0.000 (≈0.000) †	−0.001 (0.000)**	−0.009 (0.002)***	0.055 (0.006)***	0.045 (0.007)***
PH + TPH (D2)	−0.010 (0.001)***	−0.001 (0.000)***	−0.002 (0.000)***	−0.013 (0.001)***	0.093 (0.004)***	0.081 (0.004)***
PH + APH (D3)	0.045 (0.003)***	0.001 (0.000)**	0.007 (0.002)***	0.053 (0.003)***	−0.025 (0.010)**	0.029 (0.011)**
PH + TPH + APH (D4)	0.004 (0.003)	0.000 (0.000)	0.001 (0.000)	0.005 (0.003)	0.015 (0.010)	0.021 (0.010)*

* p<.05 , ** p<.01 , *** p<.001 ; † For TPH the via-MLI term rounds to 0.000 with p=.050 (BC 95% CI includes zero); no direction is interpreted.

By contrast, PH + APH exhibited a uniformly positive mediation profile. The via-MHI pathway was sizeable (0.045, *p* < 0.001), supplemented by a smaller serial effect (0.007, *p* < 0.001) and a modest via-MLI effect (0.001, *p* < 0.01). Although its direct component was negative (c′ = −0.025, *p* < 0.05), the positive mediation more than offset it, producing a positive total effect (0.029, *p* < 0.01). For PH + TPH + APH, none of the indirect components reached significance and the aggregated indirect effect was trivial (0.005, ns); the small positive direct effect (c′ = 0.015, ns) resulted in a marginal total effect (0.021, *p* < 0.05).

Bootstrapped bias-corrected 95% CIs (from Mplus) corroborated these inferences: all effects flagged as significant in Table 4 had CIs excluding zero, whereas non-significant entries included zero.

#### Pairwise contrasts among non-reference categories

Using MODEL CONSTRAINT, we compared categories directly (D1–D4). Key findings (non-standardized):

(1) Direct effects to MAP ( $c^{\prime }$ ): D2 had a larger direct effect than D1, D3, D4 (e.g., $c_{2}^{\prime }-c_{1}^{\prime }=0.039,p<.001;c_{2}^{\prime }-c_{3}^{\prime }=0.118,p<.001$ ). D1 exceeded D3 ( $c_{1}^{\prime }-c_{3}^{\prime }=0.079,p<.001$ ); D1 exceeded D4 ( 0.039,p<.001 ); D3 < D4 ( −0.040,p=.003 ).
(2) Indirect via MHI: D3 ≫ D1/D2/D4 (e.g., ${\mathrm{ind}}_{\mathrm{MHI},3}-{\mathrm{ind}}_{\mathrm{MHI},2}=0.055,p<.001$ ). D1 and D2 were more negative than others.
(3) Serial (MHI → MLI): same ordering as via-MHI; D3 greater than D1/D2/D4 (all p≤.004 ).
(4) Total indirect: D3 > D1/D2/D4 (all p≤.004 ); D1 and D2 < D4 (both p<.001 ).
(5) Total effects: D2 exceeded D3 and D4 (+0.052, p < 0.001; +0.060, p < 0.001). D1 exceeded D4 (+0.025, *p* = 0.031) but was not significantly different from D3 (+0.017, *p* = 0.155).

## Discussion

Using PH as the reference category, this study finds that all three patterns except PH + APH showed a positive direct predictive effect on students’ MAP. Mathematics homework interest and mathematics learning interest partially explain the associations between mathematics homework types and their combinations and MAP; however, the mediating effects vary depending on the specific homework types and their combinations. Additionally, they exert a serial mediating role in the relationship between mathematics homework types and their combinations, and mathematics academic performance. The results are discussed below.

### The relationship between mathematics homework types and mathematics academic performance

This study first investigated the direct associations between the types and combinations of mathematics homework and students’ mathematics academic performance under the background of China’s “Double Reduction” policy. For the first hypothesis, when using PH as the reference category, the results show that the combination of PH + TPH has the strongest predictive power for mathematics academic performance. At the same time, it is also the most common combination of mathematics homework for Chinese students, accounting for more than half of the students. Such a result is not unexpected, according to functional theories of homework (Epstein and Van Voorhis, 2001), the function of PH is to enable students to consolidate and transfer the knowledge they have learned, and the function of TPH is to prepare for exams. The combination of the two may provide students’ with opportunities for both knowledge consolidation and examination practice, which could partially explain its positive association with mathematics academic performance. However, it should be noted that high-quality homework should aim at supporting students’ learning (Trautwein, 2007), rather than merely serving exam-oriented tests. In the new national 2022 Mathematics Curriculum Standards (Ministry of Education of People’s Republic of China, 2022), it is pointed out that the examination-oriented teaching model has limitations. This curriculum standard shifts the goal of school education from “knowledge coverage” to “core literacy cultivation,” and emphasizes problem-based learning methods. From this perspective, APH is theoretically expected to provide opportunities for connecting mathematical knowledge with real-life situations, design practices, modeling activities, and inquiry processes (Park, 2022), although the homework combinations containing APH have relatively weak predictive power for mathematical academic performance. Furthermore, although PH + APH has a slight negative direct effect on mathematical academic performance compared to PH, it also has a stable indirect effect. Moreover, the addition of TPH weakens the motivational advantage, which is more profound than only mentioning the influence of homework types in the existing literature and reveals a more complex mechanism of action behind the combination. One possible explanation is that frequent exposure to exam-oriented homework may be associated with a stronger emphasis on performance goals rather than conceptual understanding, which could weaken students’ motivational engagement (Kirkpatrick and Zang, 2011; Simzar et al., 2015).

### The mediating role of mathematics homework interest

Previous studies have pointed out that homework interest should be studied as an independent construct (Cooper et al., 1998). The results of this study further demonstrate the important role of mathematics homework interest as an independent variable in students’ development. As a mediating variable, MHI has both positive and negative association with mathematical academic performance, depending on the type of mathematics homework and its combination. We found that adding APH on top of PH (PH + APH) can greatly enhance students’ mathematics homework interest,and our findings provide one possible explanation for why PH alone may be insufficient to enhance students’ homework interest: Recent analyses of eighth-grade mathematics textbooks in China have shown that in the latest curriculum reforms, the proportion of real-life scenarios contained in the textbook content has shown a slight downward trend - although such homework can help students consolidate knowledge and skills, its ability to stimulate curiosity is thus limited (Liu et al., 2022). And for TPH and PH + TPH (PH as reference), the indirect path generated solely through interest in math homework is negative, which to some extent suppresses its positive direct effect on academic performance. This result is consistent with the conclusion of classroom observations: Repetitive, training-centered exercises (such as TPH), even if they can enhance test-taking skills, may also weaken students’ pleasure with the task itself (Kaur, 2011). On the contrary, the effect of PH + APH, which is only generated by interest in mathematics homework, is positive. This is consistent with the relevant research view that tasks with novelty, relevance and autonomy (such as APH) can stimulate situational interest (Shernoff et al., 2003), and can maintain students’ intrinsic motivation for homework in the long term (Xu, 2008; Xu, 2021). Several theoretical perspectives may help interpret these differences. The first one is the transmission of task value: APH conveys to students the signal that “mathematics is not only used for problem-solving and answering questions, but also for proposing and solving practical problems,” and this kind of value cognition is precisely the core prerequisite for generating interest in homework (Suárez et al., 2019). The second is autonomy and competence support: APH usually allows students to make independent choices, participate in design, and produce perceptible specific results (such as plans, models), and such designs have a significant effect on stimulating motivation (Deci and Ryan, 2013). On the contrary, frequently assigning exam-oriented homework (TPH) conveys a “narrow performance goal” (such as being only oriented towards test scores). Although it may increase students’ learning pressure, it fails to deepen their understanding of the practicality of mathematics or enhance their learning autonomy, which may be one possible reason for the lower levels of homework interest observed among students reporting TPH (Xu et al., 2016).

### The mediating role of mathematics learning interest

Previous studies have shown that learning interest is a stable trait and is strongly correlated with academic performance (Köller et al., 2001; Hattie, 2009). However, in this study, MLI also played a mediating role in the association between homework types and mathematics academic performance, but to a relatively mild extent. Taking PH as a reference, PH + APH has a significant and positive predictive power for mathematics learning interest, PH + TPH + APH’s predictive power is also positive but not significant, while TPH and PH + TPH have a significant and negative predictive power. This asymmetry is reasonable: the textbook exercises are in line with the course progress, and long-term practice may provide opportunities for knowledge consolidation, which has been theoretically associated with the development of domain interest (Hidi, 1990; Köller et al., 2001). TPH does not have an effect solely generated by interest in learning mathematics, which is consistent with the conclusion of existing research that exam-style practice limits students’ perception of the value of mathematics to test scores, and this is not sufficient to cultivate domain interest with similar traits (Kaur, 2011). These results further illustrate the differences in the associations between homework types and academic performance through learning interest, providing new empirical support in the field of mathematics for research on the mediation of learning interest.

### The chain mediating roles of mathematics homework interest and mathematics learning interest

Our research supports the chain path of “Mathematics Homework Interest (MHI) → Mathematics Learning Interest (MLI) → Mathematics Academic Performance (MAP),” but the direction of this path also varies depending on the type of homework. Reference to PH, PH + APH produced a positive chain effect, while TPH and PH + TPH produced a negative chain effect. This sequence is highly consistent with the four-stage model of interest development (Hidi and Renninger, 2006), when students experience the repetitive cycle of “trigger → maintain → deepen” in cognitive challenges and feedback, situational interest stimulated by interesting homework can develop into more lasting individual interest. In our results, PH + APH seems to reflect such a cycle - textbook-related exercises consolidate knowledge and skills, application-related tasks maintain curiosity, thereby transforming interest in mathematics homework into interest in mathematics learning, and ultimately influencing academic performance. In contrast, excessive reliance on TPH may only trigger a short-term sense of vigilance without positive emotions accompanying it, interrupting the development process from situational interest to individual interest and thereby generating a negative chain mediating effect. This not only verifies the existence of chain mediation, but also explains the direction differences of chain mediation under different types of homework, and improves the research on the relationship and mechanism of action between homework interest and learning interest.

## Conclusion

This study explores what types of mathematics homework and their combinations are most beneficial to students’ academic development under the background of China’s “Double Reduction” policy. The results show that all five types of homework (and their combinations) have different relationships with academic development, and there are differences in the paths they take to stimulate learning motivation. Among the combinations, PH + APH showed positive mediation through the ‘via MHI’, ‘via MLI’, and ‘MHI → MLI’ routes, resulting in a positive total effect despite a small negative direct component. This result indicates that real applied tasks were associated with higher homework interest and mathematics learning interest, and providing support for academic performance.

On the contrary, if the educational goal is set to maximize the improvement of exam scores, then PH + TPH is the optimal combination, because the combination containing TPH still retains a positive direct effect. However, it should be noted that: the indirect effect generated through the interest in mathematics homework (MHI) is negative - this is consistent with the inherent, training-like and test-oriented characteristics of exam-oriented homework. These results supplement the detailed mechanism-level findings in the research literature related to “homework - performance”: Meta-analysis studies have confirmed that the overall benefits of homework are usually limited and depend on the way students actually complete their homework (Fan et al., 2017), and the results of this study further expand this evidence; Meanwhile, these findings are also consistent with the conclusions of multi-level studies in specific fields, that is, such studies emphasize that homework design and learning engagement are the direct key factors influencing academic outcomes (Trautwein et al., 2006).

Overall, the research results support that homework design should shift from “more homework” to “better combinations”: combining PH with APH, while selectively using TPH only for diagnostic purposes. In this way, it can not only promote the development of students’ abilities but also maintain good academic performance.

### Theoretical and practical implications

Theoretically, the data specify how homework works: task-proximal MHI appears to be the entry point that cultivates broader, trait-like MLI, which then supports mathematics academic performance. This sequencing clarifies why applied tasks—through relevance, choice, and context—can energise both forms of interest, whereas pure exam practice may yield short-term score gains while weakening motivational pathways. The asymmetric pattern we observe reconciles mixed prior conclusions by distinguishing direct performance effects from interest-mediated effects (Fan et al., 2017). Practically, schools—the basic units of reform—can act immediately without increasing workload: prioritize the PH + APH pairing as the default homework combination; use TPH sparingly and strategically; and embed small design features in PH (brief reflection, real-world hooks, limited choice) to offset its weaker interest signal. Given evidence that textbook problem sets have become less context-rich (Liu et al., 2022), APH can provide the embodied, experiential problem-solving skills emphasized by the curriculum standards and restore the motivational value of homework.

### Limitation and direction for future research

This study still has some limitations. For instance, it only focuses on the association between different types and combinations of homework and academic performance and their underlying mechanisms, and emphasizes the mediating roles played by homework interest and learning interest. However, there is still a lack of further exploration in some variables related to homework. For example, Our study found a strong positive correlation between homework interest and learning interest (r = 0.771), indicating that the two constructs are closely intertwined in practice. Although our model treats them as distinct links in a sequence, it is also possible that they mutually reinforce and develop together. Some studies have pointed out that the time students invest in their homework, their attitude towards it and the quality of completion all affect the benefits produced by the homework (Fan et al., 2017). This study has not yet systematically integrated these variables. For instance, the role played by teachers in enhancing students’ interest in homework is significant. How teachers select homework themes and contents that are in line with the curriculum standards (Trautwein et al., 2006), how they participate in the process of students completing homework, and how they evaluate the completed homework all require further clarification in subsequent studies. In addition, two methodological limitations should be noted. First, school-level identifiers were unavailable, which prevented us from applying multilevel modeling; future studies with such data could examine cross-level interactions. Second, the cross-sectional design limits causal inference; future work using cross-lagged panel models could better determine the directionality of the relationships.

## Data Availability

The datasets analyzed in this study are not publicly available. The data were collected as part of a funded research project and are subject to institutional and contractual restrictions. Due to data protection regulations and agreements with participating institutions, the raw data cannot be shared publicly or upon request. Access to the data is restricted to authorized members of the research team. Any inquiries about the dataset can be directed to the corresponding author/s.
